# Diagnosis of human angiostrongyliasis in a case of hydrocephalus using next-generation sequencing: a case report and literature review

**DOI:** 10.1186/s12883-024-03663-7

**Published:** 2024-08-12

**Authors:** Dayuan Liu, Ning Li, Yubo Zhu, Qianhua Chen, Xudong Fan, Jigao Feng

**Affiliations:** 1 Department of Neurosurgery, The Second Affiliated Hospital of Hainan Medical University, 368 Yehai Avenue, Longhua District, Haikou City, Hainan Province 570311 China; 2 Hainan Medical University, No.3 Xueyuan Road, Longhua District, Haikou City, Hainan Province 571199 China

**Keywords:** Metagenomic next-generation sequencing, *Angiostrongylus cantonensis*, Eosinophilic meningitis, Diagnosis

## Abstract

**Background:**

Angiostrongyliasis cantonensis is a severe yet rare parasitic infection caused by the larvae of *Angiostrongylus cantonensis*. The primary characteristic feature of this foodborne illness in humans is eosinophilic meningitis. Recently, there has been a gradual increase in reported cases globally. Due to the lack of typical clinical symptoms, signs, and specific laboratory tests, early diagnosis of this disease poses significant challenges. Failure to diagnose and treat this condition promptly can result in fatalities.

**Methods:**

We present the case of a 13-year-old male patient who initially presented with fever and headache. The patient was preliminarily diagnosed with bacterial meningitis and received treatment with antibacterial drugs. However, the patient’s condition worsened, and he developed progressive consciousness disturbances. Eventually, metagenomic next-generation sequencing (mNGS) testing of cerebrospinal fluid samples indicated *Angiostrongylus cantonensis* infection. Following treatment with albendazole and prednisone, the patient made a full recovery. We include this case report as part of a literature review to emphasize the potential applications of mNGS in the early diagnosis of Angiostrongyliasis cantonensis.

**Conclusion:**

mNGS technology plays a crucial role in the diagnosis of angiostrongyliasis cantonensis. As this technology continues to evolve and be applied, we believe it will play an increasingly important role in diagnosing, treating, and monitoring angiostrongyliasis cantonensis.

## Introduction

Rat lungworm, identified as *Angiostrongylus cantonensis* (*A. cantonensis*) by Dougherty in Guangzhou in 1946, was discovered in China in 1993 [1]. Angiostrongyliasis cantonensis is a newly discovered parasitic disease that is caused by the third-stage larvae of the *A. cantonensis*, which parasitizes the human body and primarily presents as eosinophilic meningitis (EM) [1]. Consuming raw or undercooked intermediate hosts, such as snails or pomacea canaliculata, and contaminated vegetables or fruits poses a risk for infection. Notably, meningitis and encephalitis caused by *A. cantonensis* represent a rare category of diseases whose clinical symptoms lack specificity compared to other intracranial infectious diseases [2]. Furthermore, the clinical presentation varies significantly depending on factors such as age, geographical region, and exposure mode among infected individuals, often leading to misdiagnosis and serious consequences [3]. Meningoencephalitis or meningitis caused by *A. cantonensis* often presents as dizziness, headaches, nausea, vomiting, fever, seizures, and other clinical symptoms associated with increased eosinophilic granulocytes [4]. In severe cases, some patients may experience coma, decerebrate rigidity, and even death [5, 6]. Delayed diagnosis is common due to the absence of typical or specific clinical symptoms and biological markers in its early stages, combined with limited awareness and diagnostic capabilities among clinical practitioners regarding this rare disease [5]. Therefore, early diagnosis is vital in guiding treatment, reducing complications, and lowering mortality rates.

Early diagnostic methods for this disease include blood tests, cerebrospinal fluid analysis, qPCR, and brain magnetic resonance imaging (MRI). However, the success rate of these tests is often unsatisfactory, mainly when the worm burden is low. Additionally, only 70% of patients have an elevated eosinophil count in their cerebrospinal fluid, and detection rates are even lower in the early stages of the disease [7]. Here, ‘early stages’ refers to the initial phase after the onset of symptoms due to *Angiostrongylus cantonensis* infection. Precisely, it is within the first week of symptom manifestation, which is typically before seroconversion where we see a significant IgM or IgG response. Unfortunately, this poses further difficulty in diagnosis [8]. In the early stages of infection, there may be no abnormal elevation of eosinophil levels or positive antibody test results [9, 10]. Thus, many cases in the early stage are misdiagnosed as viral encephalitis, Guillain-Barré syndrome, or bacterial meningitis due to the lack of a definitive diagnosis [11]. This not only wastes healthcare resources but also causes additional suffering for patients.

Recently, there have been increasing reports on using metagenomic next-generation sequencing (mNGS) for early diagnosis of *A. cantonensis* infections [12–14]. Herein, we report a case of a 13-year-old boy initially diagnosed with viral encephalitis complicated by hydrocephalus. However, subsequent cerebrospinal fluid mNGS testing revealed *A. cantonensis* infection, leading to an accurate diagnosis and successful treatment. Thus, this case emphasizes the importance of mNGS in the early diagnosis of *A. cantonensis* infections.

## Case report

A 13-year-old male patient was admitted to the emergency department with a 3-day history of headache, vomiting, and fever. He initially presented with fever, followed by dizziness, headaches, and nausea, which did not improve with empirical antibiotic treatment at a local hospital (Lingshui County, Hainan Island, located in southern China). As his condition worsened, he developed confusion and was urgently transferred to our hospital for treatment.

Upon physical examination, the patient was confused with decreased cognitive ability, spontaneous eye-opening, dysarthria, and withdrawal in response to pain. Additionally, the Glasgow Coma Scale (GCS) score was 9 [14]. Neck stiffness and positive Kernig’s and Brudzinski’s signs indicated meningeal irritation, and there was increased muscle tone in all limbs. However, no pyramidal tract signs such as Babinski’s, Hoffman’s, or Oppenheim’s were observed. Furthermore, the patient had an intermittent fever (37.5 –39.5 °C).

The patient had no significant medical history, received vaccinations according to the recommended schedule, and denied any immunodeficiency disorders or recent infections in other body parts.

**Fig. 1:**
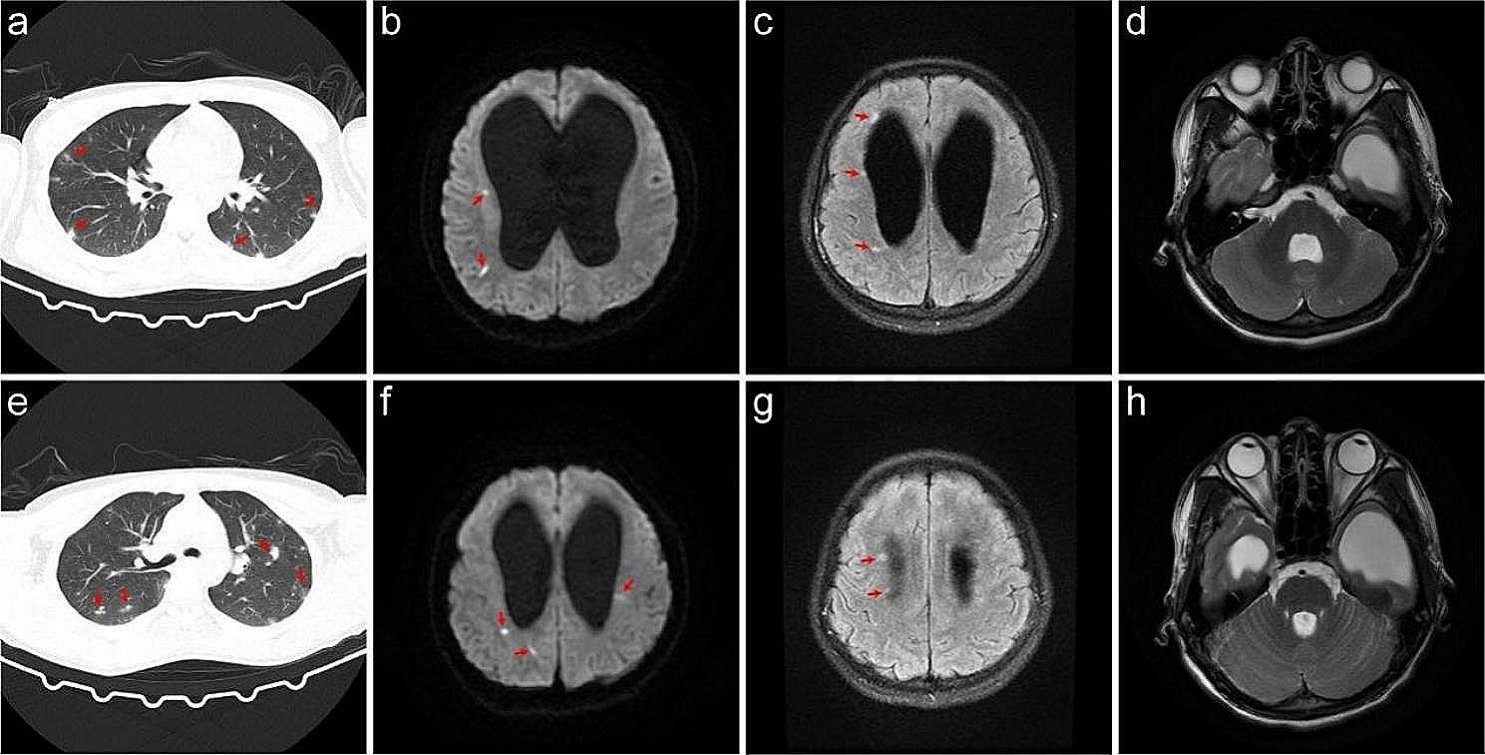
Imaging features of the patient with severe CNS angiostrongyliasis. **A** & **e** Chest CT scan reveals diffuse nodules and infiltrates in bilateral lung lobes; **b** & **f** MRI DWI sequence reveals scattered high signal intensity lesions in the brain parenchyma and around the ventricles; **c** & **g** MRI axial FLAIR sequence shows scattered high signal lesions around the ventricles; **d** & **h** MRI axial T2 sequence shows the fourth ventricle and the temporal horns of bilateral ventricles are significantly dilated

Upon admission, laboratory tests revealed that the complete blood cell count was within normal range, while procalcitonin (PCT) levels were mildly elevated at 0.47 ng/ml. During the initial lumbar puncture procedure, an opening pressure of 300 mmH_2_O was observed, and cerebrospinal fluid (CSF) appeared clear with normal glucose (3.36 mmol/L) and chloride (121.0 mmol/L) levels. Additionally, total protein content was slightly elevated at 552.65 mg/L with positive for the Pandy test, white blood cell count was 147.00 × 106/L, and the percentage of mononuclear cells was increased to 95%. Staining of CSF revealed no bacteria, fungi, Cryptococcus, or acid-fast bacilli. The pulmonary computed tomography (CT) examination showed both lungs’ patchy and nodular high-density shadows (Fig. 1a & Fig. 1e). Moreso, a head MRI examination revealed severe hydrocephalus (Fig. 1d & Fig. 1h) and abnormal high-signal punctate lesions adjacent to the bilateral lateral ventricles on T2-weighted Fluid-Attenuated Inversion Recovery (T2FLAIR) (Fig. 1c &Fig. 1g) and Diffusion-Weighted Imaging (DWI) sequences (Fig. 1b & Fig. 1f). The patient was initially diagnosed with viral meningitis, and his condition continued deteriorating. The patient’s fever persisted, and their consciousness disorder further worsened. Intermittent lumbar puncture measurements consistently showed intracranial pressure (ICP) above 300 mm H_2_O. On the fourth day following admission, the patient was transferred to the Neurosurgical Intensive Care Unit (NICU) for treatment and underwent tracheal intubation. The patient had a persistent fever greater than 39.0℃ and was treated with sub-hypothermic brain protection therapy [15, 16]. On the seventh day of hospitalization, Metagenomic Next-Generation Sequencing (mNGS) was employed for pathogen detection in the cerebrospinal fluid. The mNGS (Hugobiotech, Beijing, China) was performed as described. DNA extraction was performed using the QIAamp DNA Micro Kit (QIAGEN). Subsequently, the library was constructed utilizing the QIAseqTM Ultralow Input Library Kit (Illumina, San Diego, CA, USA). The quality of the library was then assessed via Qubit (Thermo Fisher), and an Agilent 2100 Bioanalyzer (Agilent Technologies, Santa Clara, CA, USA), before being sequenced on a NextSeq 550 platform (Illumina, San Diego, CA, USA). We removed short, low-quality, and low-complexity reads from the raw data and also filtered out human DNA by aligning it to the human reference database (hg38) using Bowtie 2. Finally, the remaining reads were aligned to the Microbial Genome Databases (http://ftp.ncbi.nlm.nih.gov/genomes/) using the Burrow-Wheeler Aligner (BWA). In addition, a routine blood test revealed that the white blood cell count in the peripheral blood was 13.93 × 10^9^/L, with an increased eosinophil count (41.0% proportion and 5.71 × 10^9^/L absolute count). Three days later, *A. cantonensis* was reported by mNGS from the CSF sample.

**Fig. 2:**
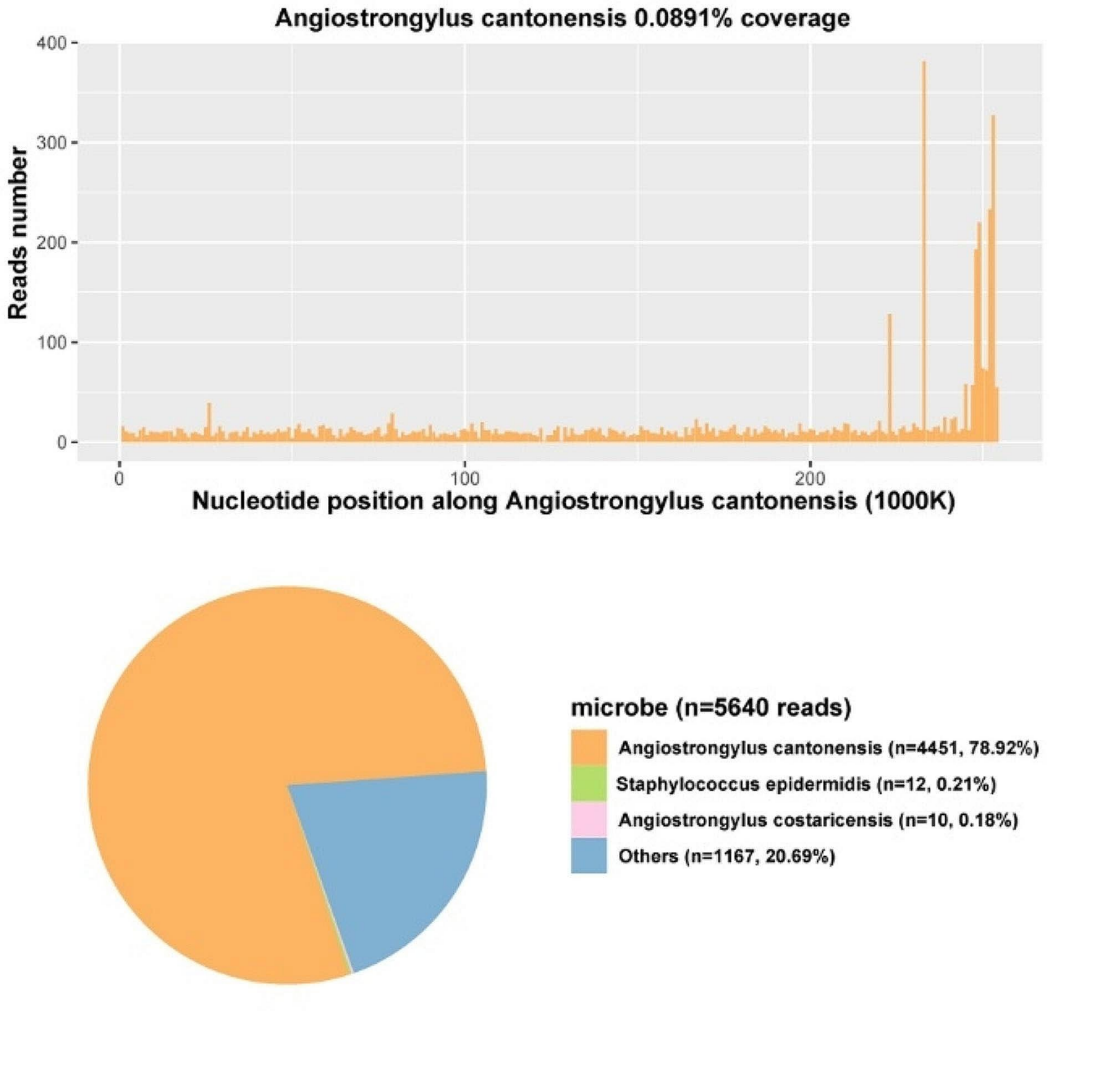
The cover charts of CSF mNGS for diagnosis

A total of 4,451 unique reads mapped to the *A. cantonensis* genome out of 5,640 microbial reads with a genome coverage rate of 0.0892%. (Fig. 2). Upon further inquiry into the patient’s medical history, it was discovered that the patient had consumed Giant African snails more than ten days before developing symptoms. Following the mNGS results, the patient was started on a standard treatment regimen of albendazole (400 mg twice daily) and prednisone (60 mg once daily). Due to persistent intracranial hypertension (ICP ≥ 300 mmH_2_O) and hydrocephalus, the patient underwent an external ventricular drainage procedure on the 12th day after the onset of symptoms, which continued for 14 days before removing the external ventricular drain.

**Fig. 3:**
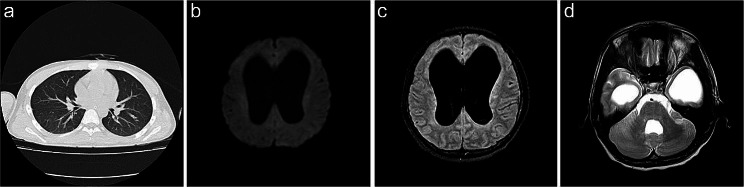
Imaging follow-up after six months of treatment reveals the disappearance of lesions in the lungs and brain. **a** epresents the chest CT scan; **b** represents the axial DWI sequence of MRI;**c** represents the T2Flair sequence of MRI; **d** represents the T2 sequence of MRI

The patient received continued treatment with albendazole and prednisone for an additional 30 days, during which clinical symptoms gradually improved. The patient’s body temperature gradually returned to normal, and the peripheral blood eosinophil count decreased gradually to within the normal range. Consciousness became increasingly clear, and the patient achieved a GCS score of 15 with no residual neurological deficits. Furthermore, head MRI and pulmonary CT scans conducted for follow-up six months later revealed a significant regression of the lesions. However, there is no significant reduction observed in the bilateral ventricles and the 4th ventricle of the patient, and there are no interstitial brain edema changes in the brain parenchyma (Fig. 3). Importantly, prior to this episode, the patient did not exhibit any symptoms or signs suggestive of increased intracranial pressure, nor were there anomalies detected in cognitive development. In light of these observations, it is incumbent upon further inquiry to ascertain whether the observed hydrocephalus is of congenital origin or a complication ensuing from the recent infection. The patient was subsequently discharged and received further rehabilitation training.

## Discussion and conclusions

Angiostrongyliasis cantonensis is a rare but serious disease that can lead to eosinophilic meningitis [2]. While this condition is commonly believed to be self-limiting, cases of residual neurologic sequelae and mortality are not uncommon [17]. Therefore, early diagnosis and treatment are crucial for reducing eosinophilic meningitis’s mortality and morbidity rates. The diagnostic criteria for angiostrongyliasis cantonensis are the presence of *A. cantonensis* larvae or developing adults in the CSF or eye, although the detection rate is often low in clinical practice [18]. In this case, we demonstrate that mNGS is an effective method for the early diagnosis of angiostrongyliasis cantonensis.

The current clinical diagnostic methods for angiostrongyliasis cantonensis mainly include typical clinical manifestations, positive detection of specific antibodies or circulating antigens of *A. cantonensis* in serum or CSF, elevated eosinophil count in blood and CSF, as well as brain and spinal cord MRI examination [19]. Consequently, infection with *A. cantonensis* in humans can cause an increase in eosinophil count in the CSF (although the exact mechanism is unclear), activate the central nervous system’s inflammatory response, and lead to encephalitis or meningitis through the synergistic action of various cytokines and chemokines (such as IL-5, IL-33, and IL-1b) [7, 8]. Most patients experience an initial increase in peripheral blood eosinophil counts at the onset of infection, which gradually decreases following treatment and alleviation of the disease, typically returning to normal within three months [20]. However, there have also been cases where no increase in eosinophil count was observed in the CSF or blood [21–23]. Chaiwun et al. also reported a case of a patient with myelitis caused by *A. cantonensis* infection, which solely involved the spinal cord. In this case, the patient was misdiagnosed with a spinal cord tumor and underwent surgical resection. Postoperative histopathological examination confirmed the presence of *A. cantonensis*, with inflammation mainly surrounding the parasite composed of lymphocytes, plasma cells, and a few eosinophils. While eosinophil count can be a valuable diagnostic clue, it is not always present and may be influenced by the degree of peripheral blood eosinophilia [24]. Our case presented a significant challenge for early diagnosis of eosinophilic meningitis since an increase in peripheral blood eosinophil count was only observed on the seventh day of hospitalization (the 10th-day post-infection). Notably, Hsueh et al. reported a case of a 15-year-old male patient with *A. cantonensis* infection who only exhibited urinary retention. The patient had no fever, headache, or other symptoms of intracranial infection, and brain and spinal cord MRI did not reveal any abnormalities [25].In the early stages, specific antibody detection in serum and CSF usually has a low positivity rate [26]. Cases with atypical clinical symptoms can pose a significant diagnostic challenge at the early stages of onset, particularly in non-endemic regions where physicians may have limited experience with the disease. Radiological examinations are not routinely used to diagnose eosinophilic meningitis caused by *A. cantonensis* infection [3]. Some scholars believe that most patients have normal results on head CT or MRI scans and that radiological features are diverse and lack specificity [27–29]. However, radiological examinations can aid in early diagnosis and help predict prognosis by identifying lesion location and degree of brain tissue damage [30]. In addition, CT or MRI can distinguish *A. cantonensis* infection from other parasitic diseases. For example, eosinophilic meningitis caused by *A. cantonensis* infection is characterized by meningeal enhancement, abnormal cerebral enhancing lesions, and T2 hyperintense lesions [19]. In contrast, eosinophilic meningitis caused by *Gnathostoma spinigerum* infection can lead to hemorrhagic changes [19, 31–33]. There have also been reports of cases with concurrent hydrocephalus treated with ventriculoperitoneal shunting. It has been suggested that hydrocephalus is associated with a worse prognosis, as it can lead to an increase in intracranial pressure. If this is not promptly and effectively managed, it could result in significant neurological implications such as cognitive impairment or loss of motor function [17, 34]. Spinal cord involvement is also common and typically presents as T2 hyperintense signals within the spinal cord [28, 35, 36]. Some scholars have reported MRI features, including periventricular and deep gray matter hyperintensity on T2/T2-FLAIR, abnormal hyperintensity on diffusion-weighted imaging (DWI), and mild lesion enhancement, consistent with the imaging characteristics of our case report [30, 37]. Tsai et al. found that there was a correlation between the degree of lesion enhancement on brain MRI and the number of eosinophils in blood/CSF, as well as the detection rate of *A. cantonensis* antibodies in CSF, based on clinical and radiographic features from 26 patients [38]. However, the role of cranial MRI in the diagnosis and prognosis evaluation of angiostrongyliasis cantonensis requires additional investigation.

The mNGS detection of CSF has begun to be applied in the diagnosis and treatment management of infectious, neoplastic, immune-mediated, and degenerative diseases of the central nervous system [39], especially in the diagnosis of infectious encephalitis/meningitis, where it is superior to conventional methods [40]. It has also emerged as a promising diagnostic tool for the early detection of angiostrongyliasis cantonensis. This approach demonstrates high sensitivity and specificity, faster turnaround times, and the ability to detect multiple pathogens simultaneously compared to conventional diagnostic modalities, including serology and microscopy [41]. These unique features make mNGS a powerful diagnostic technique for eosinophilic meningitis caused by *A. cantonensis*, particularly in non-endemic areas where physicians may have limited experience with this disease [12]. Our study summarized the case characteristics of our own and other cases using mNGS to diagnose Angiostrongyliasis cantonensis (Table 1). The first and second cases showed *A. cantonensis* infection detected by mNGS in the early stages of the disease. However, CSF-specific antibody ELISA only became positive on days 35 and 31 post-infection, respectively, significantly later than the mNGS diagnosis. Prompt treatment with albendazole deworming and corticosteroid therapy led to complete recovery. In contrast, the third and fifth cases were diagnosed with *A. cantonensis* infection by mNGS in the early stages of the disease. At the same time, their CSF-specific antibody test results were negative. Moreso, it can be seen that the specific DNA sequence read numbers vary significantly among different cases. When other microorganisms are present, the read numbers for *A. cantonensis* detected by mNGS tend to be larger. In contrast, the DNA-specific sequence read numbers for cases with only *A. cantonensis* infection are smaller. This may be useful as a reference when interpreting mNGS results in clinical settings. Meanwhile, studies have found that the strictly mapped reads number (SMRN) of *A. cantonensis* DNA in CSF detected by mNGS is positively correlated with lumbar puncture opening pressure and clinical manifestations, indicating that mNGS may serve as an essential indicator for assessing disease progression, treatment effectiveness, and prognosis evaluation [11, 26]. It’s worth noting that when using mNGS for microbial diagnostics, multiple pathogenic microbes might be detected. The choice of which one to consider as the primary pathogen should be made in conjunction with clinical presentations and other laboratory test results to avoid overinterpretation. In this case, besides *A. cantonensis*, *Angiostrongylus costaricensis* and *Staphylococcus epidemidis* were also detected. However, *Angiostrongylus costaricensis* primarily causes intestinal diseases and its unique reads were significantly lower compared to *A. cantonensis* (4451 versus 10). Bacterial meningitis caused by *Staphylococcus epidemidis* infection typically results in biochemical abnormalities in the CSF, such as an increase in white blood cell count and protein content, while glucose and chloride levels usually decrease. However, in this instance, both glucose (3.36 mmol/L) and chloride (121.0 mmol/L) in the patient’s CSF were within the normal range, thus both were ruled out.

**Table 1:** Summary of CSF mNGS features in cases of *A. cantonensis* infection

Study	Sex/age	CSF Eosinophilia (%)	ELISA	SSRN reads	Coverage(%)	Other Microbe	Confirmatory data
Liu et al. 2022 [11]	M/8	NA	+	22	0.0079	No	PCR, antibody
Feng et al. 2020 [9]	M/27	67	+	13,492	0.3115	Yes	qPCR, antibody
Zou et al. 2020 [21]	M/43	80	-	3805	0.0645	NA	Clinical
Xie et al. 2019 [10]	M/2	68.91	+	3204	0.0786	No	third-stage larva was found in ocular
	M/3	33	-	892	0.0345	No	Clinical
Zhang et al. 2019 [12]	M/59	21.9	+	17,202	NA	Yes	Antibody, Clinical
This study	M/13	NA	NA	4451	0.0892	Yes	Clinical

SSRN species-specific read number, NA not available, ELISA enzyme-linked immunosorbent assay: The results of Elisa revealed that both the serum and the CSF were positive forantibodies against *A. cantonensis*, Clinical evidence: patient’s history, clinical presentation, imaging finding, routine laboratory CSF results and response to antibiotic treatment. PCR polymerase chain reaction, qPCR quantitative polymerase chain reaction

Of note, the high cost and technical complexity of mNGS may restrict its widespread application in resource-constrained environments [42, 43]. Currently, mNGS’s comprehensive cost encompasses multiple stages, including sample preparation, sequencing, and data analysis. For regions with limited resources or patients under financial constraints, such expenses may pose significant burdens. Furthermore, the high technical requirements of mNGS, demanding precise operations and advanced equipment, could amplify the complexity and risk inherent in the experiments. Moreover, the precision and reliability of mNGS call for further validation across diverse populations and geographical locales. Due to the variations in geography, genetic backgrounds, and environmental factors, the types and distribution of pathogens might differ among distinct populations. Despite these existing challenges, we remain confident that ongoing advancements in technology coupled with deepening research will result in eventual solutions. For instance, the advent of new sequencing platforms and improved data processing methods may potentially decrease both the cost and complexity associated with mNGS. Concurrently, by conducting large-scale studies across varied populations, we can foster a better understanding of and response to geographical and genetic disparities among pathogens. It is worth noting that manufacturers have now designed targeted Next-Generation Sequencing (tNGS) panels for diagnosing Central Nervous System (CNS) infections via CSF testing, including a wide spectrum of parasites. In contrast to the broad coverage and unbiased nature of mNGS, tNGS employs a targeted amplification or capture approach that enables the detection of a set of pathogens, gene subsets, or specific genomic regions of interest. This method typically boasts higher sensitivity and specificity, while also being more cost-effective. In conclusion, mNGS shows great promise for the early and accurate diagnosis of angiostrongyliasis cantonensis. However, additional investigations are required to establish its clinical utility and feasibility in diverse populations for diagnosing angiostrongyliasis cantonensis. What’s more, it t is imperative to clarify that mNGS may not be necessary in all cases of CNS infections. Indeed, its utility predominantly surfaces when traditional diagnostic methods fail or when dealing with pathogens that are difficult to culture or identify.

Therefore, while mNGS certainly offers a powerful tool for diagnosing CNS infections, its application should be judicious, carefully considering factors such as the suspected pathogen, available resources, clinical context, and the potential benefits and drawbacks of the test. It remains paramount to tailor diagnostic strategies to suit the individual patient’s condition and healthcare context effectively.

Angiostrongyliasis cantonensis is a rare but often fatal parasitic disease of the central nervous system that can be easily misdiagnosed as bacterial or viral encephalitis in the early stages, leading to delayed treatment [1]. This highlights the importance of considering other uncommon causes besides the common bacterial or viral infections when dealing with patients suspected of having meningitis, such as *A. cantonensis* infection, especially in patients who exhibit poor response to antibiotic therapy and marked eosinophilia [11, 44]. Notably, mNGS has many advantages, such as high sensitivity, specificity, and throughput, and can be used as an important supplement to diagnosis, shortening the time for pathogen detection and diagnosis and reducing the rates of misdiagnosis and missed diagnosis [45]. Thus, it is expected that mNGS will be widely used in the diagnosis, treatment, and prognosis evaluation of CNS infectious diseases.

## Data Availability

The datasets generated and/or analyzed during the current study are available in the SRA database, https://www.ncbi.nlm.nih.gov/sra/PRJNA1092367.

## Abbreviations

mNGS: Metagenomic next-generation sequencing
*A. cantonensis*: *Angiostrongylus cantonensis*
EM: Eosinophilic meningitis
MRI: Magnetic resonance imaging
CT: Computed tomography
CSF: Cerebrospinal fluid
T2FLAIR: T2-weighted Fluid-Attenuated Inversion Recovery
DWI: Diffusion-Weighted Imaging
tNGS: Targeted Next-Generation Sequencing
CNS: Central Nervous System
